# LED Illumination for High-Quality High-Yield Crop Growth in Protected Cropping Environments

**DOI:** 10.3390/plants10112470

**Published:** 2021-11-16

**Authors:** Md Momtazur Rahman, David Luke Field, Soyed Mohiuddin Ahmed, Md Tanvir Hasan, Mohammad Khairul Basher, Kamal Alameh

**Affiliations:** 1 School of Science, Edith Cowan University, 270 Joondalup Drive, Joondalup, WA 6027, Australia; d.field@ecu.edu.au (D.L.F.); mkbasher@our.ecu.edu.au (M.K.B.); k.alameh@ecu.edu.au (K.A.); 2 School of Pharmacy & Life Science, Jiujiang University, Jiujiang 332000, China; mohiuddinppqc@yahoo.com; 3 Department of Electrical and Electronic Engineering, Jashore University of Science and Technology, Jashore 7408, Bangladesh; tan_vir_bd@yahoo.com

**Keywords:** light quality, quality crops, greenhouse, growth and development, phytochemicals

## Abstract

Vegetables and herbs play a central role in the human diet due to their low fat and calory content and essential antioxidant, phytochemicals, and fiber. It is well known that the manipulation of light wavelengths illuminating the crops can enhance their growth rate and nutrient contents. To date, it has not been easy to generalize the effects of LED illumination because of the differences in the plant species investigated, the measured traits, the way wavelengths have been manipulated, and the plants’ growing environments. In order to address this gap, we undertook a quantitative review of LED manipulation in relation to plant traits, focusing on vegetables and herbs. Here, we use standardized measurements of biomass, antioxidant, and other quantitative characteristics together with the whole range of the photosynthetic photon flux density (PPFD). Overall, our review revealed support for the claims that the red and blue LED illumination is more reliable and efficient than full spectrum illumination and increases the plant’s biomass and nutritional value by enhancing the photosynthetic activity, antioxidant properties, phenolic, and flavonoids contents. Although LED illumination provides an efficient way to improve yield and modify plant properties, this study also highlights the broad range of responses among species, varieties traits, and the age of plant material.

## 1. Introduction

Light is crucial for plant development and protection, as it provides the required energy for the photosynthesis process [1] and critical sensory signals that enable plants to adapt to external stimuli [2]. Photosynthetically active radiation (PAR) is light of wavelengths in the range 400~700 nm and is the portion of the light spectrum used by plants for photosynthesis [3]. In principle, since light-emitting diodes (LED) illumination provides PAR that the plant uses for photosynthesis, thus PAR plays a significant role in morphogenesis [4,5]. 

Moreover, the use of LEDs for greenhouse illumination has significantly improved plant quality, especially in geographic regions with reduced sunlight during winter seasons [6]. This also boost plant productivity due to the high efficiency of variable-power LEDs that can emit wavelengths over a broad spectrum. It enables the optimization of the illumination spectrum for specific plants, thus maximizing the yield and nutritional quality of the vegetables and herbs. Vegetables-based nutrition is vital for a healthy human life due to the low fat and vitamins (e.g., vitamin C), bioactive molecules (e.g., flavonoids), and fiber contents of vegetables. Herbs are also a source of phytochemicals and bioactive molecules (e.g., flavonoids) that have demonstrated potential medicinal benefits, including anti-inflammatory, anti-carcinogenic and anti-bacterial qualities [7]. Therefore, it is crucial to understand the optimal conditions of lights suitable to individual plant species and a trade-off between the plant’s yield and production costs.

Understanding optimal LED manipulation is particularly important for maximizing the yield of vegetables and herbs. Several light spectra have been trialed for enhancing the yields of vegetables and herbs grown in indoor environments. A common approach has been widely investigated to examine the effects of photosynthetic photon flux density (PPFD) on the LED illumination spectrum [8,9]. The PPFD terminology has been used in the paper to quantify the light quality, as it is defined as the number of photosynthetically active photons that fall on a given plant surface each second (Table 1). 

It has been widely accepted that the blue (417~450 nm) and red (630~680 nm) wavelength ranges are particularly effective for improving the photosynthesis process [10,11]. Interestingly, despite having lower energy (~1.82 eV), red-light wavelengths are the most effective for stimulating the photosynthetic processes [12]. Red light alone is not always favorable for plant development because of the “red light syndrome” [13]. However, these symptoms can be suppressed by adding blue light to the illumination source [14]. For example, a blue-dominated LED light source with a 440 nm peak wavelength (100 µmol m^−2^ s^−1^) has been shown to increase the pigment concentration in plants [15].

Green and far-red LED illumination can also positively impact plant growth and development among the other light spectra. For example, a green LED light can penetrate deeper into the leaf than blue and red LED illumination, thus increasing carbon fixation and improving the plant yield [16,17]. Moreover, a blue light-induced stomatal opening can be overturned using green LED illumination [18]. Similarly, far-red LED illumination has been shown to be pro-active in enhancing photosynthesis due to its synergetic effect [19]. However, by studying growth scenarios in some plants, reduction in nutrient contents has been observed when far-red light is used as a supplemental with red light [20], whereas, in other scenarios, far-red supplemental resulted in positive effects on plant growth [21]. 

Recently, moderate-intensity UV light has been found to improve the photosynthesis process, primarily if photo-protective molecules, such as flavonoids, are used, which significantly absorb ultraviolet A (UV-A) and ultraviolet B (UV-B) wavelengths, thus fighting against the harmful effects of UV [22]. Therefore, careful selection of the plant illumination spectrum can significantly affect the plant’s nutrient contents [23,24] and thus help develop protection schemes. 

Many studies using different LED manipulation across different plant species with wide morphological diversity and development profiles could provide opportunities for comparative analyses to investigate general patterns for plant responses to specific wavelength manipulations. Given the diverse range of plant species of plant responses and interactive effects on plants traits available, the variability in individual species responses is a crucial step in optimizing plant growth for LED based protected cropping environment. Comparative analyses of the literature are best undertaken using studies with standardized measurements of biomass. However, a comprehensive analysis of the impact of LED manipulation on plant growth must also include a quantitative assessment of the whole range of the PAR. 

Fraction of photosynthetically active radiation (FPAR), an essential index for evaluating yields and biomass production, is key to crop management. However, the shortage of suitable hyperspectral data frequently hinders accurate and reliable PAR assessments [25]. Despite numerous studies on identifying the essential wavelength ranges on greenhouse-grown vegetable and herb growth, there are no systematic reviews of the effect of the whole range of the PAR spectrum of LED illumination. This research gap is especially evident in the growth rate and nutrient contents of vegetables and herbs. In addition to the combination of optimal wavelengths of light across the PAR range, responses of vegetables and herbs may differ according to the time at which the plant was grown and which specific traits have been measured (e.g., wet or dry mass, phenolics or growth rate). Therefore, comparative studies need to consider the whole range of the PAR spectrum while controlling the experimental conditions related to when measurements are collected. One approach to achieve this is by adopting a standardized measure, such as using the Planck’s-Einstein equation and the ASTM G173-03 Reference Spectra for the range of 400~700 nm.

Therefore, this paper reviews the literature to identify the most critical aspects of LED lighting installation and explicitly organizes the comparative analysis of plant traits such as growth rate, morphology, and nutritional contents.

## 2. LED Illumination

**Figure 1:**
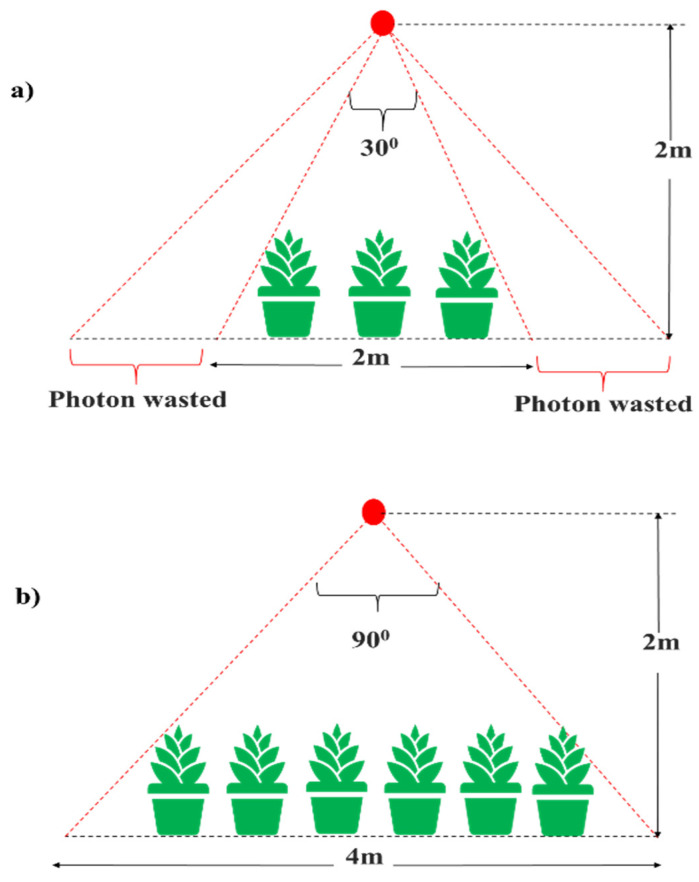
Impact of the PPF uniformity on the canopy photon capture efficiency. The canopy’s photon capture efficiency (**a**) decreases as the lighting’s fixture gets smaller and (**b**) increases as the installation of lighting gets more prominent. This concept and picture are modified [30].

Since LED illumination uniformity is essential, it prevents photons from being spread over, thus reducing the wasted electricity. Before illuminating the plant with LED sources, it is necessary to properly design the LED setup in order to ensure uniform light distribution, i.e., constant photosynthetic photon flux (PPF). For example, the canopy photon capture efficiency decreases as the lighting field become smaller (Figure 1). Moreover, the power consumption tends to increase with greenhouse size due to the inadequate arrangement of the LED lighting system. This results in nonuniform photosynthetic photon flux (PPF) distribution or a smaller plant growth area under the fixture, thus leading to wasted radiation (i.e., electricity). Once the LED lighting system’s positioning has been optimized, the next important step is to determine the essential wavelength ranges that affect the plant growth rate and understand their interaction with the plant biology system. A plant’s biology system typically consists of primary photoreceptors, phytochromes (PHY), phototropin (PHOTO), and cryptochromes (CRY). PHYs typically capture light effectively in the red (600~700 nm) and far-red (700–800 nm) ranges [26]. In contrast, the PHOTOs, CRYs, and zeitlupe (ZLT) photoreceptors perceive light actively in the blue (400~500 nm) and the UV-A (315~400 nm) regions [27]; on the other hand, the UVB-Resistance locus 8 (UVR8) photoreceptors absorb light strongly in the UV-B (280~315 nm) regions [28,29]. Therefore, plant detailed biological features, such as photoreceptors and their absorption in the LED illumination spectrum, must be carefully considered for optimal plant growth in greenhouse environments.

The next important step is to quantify the effect of the LED illumination spectrum and the critical wavebands and their interaction with the plant system. This requires the identification of the best wavelength ranges and the impact of photosynthetic photon flux that leads to higher productivity in the greenhouse-grown plant.

**Figure 2:**
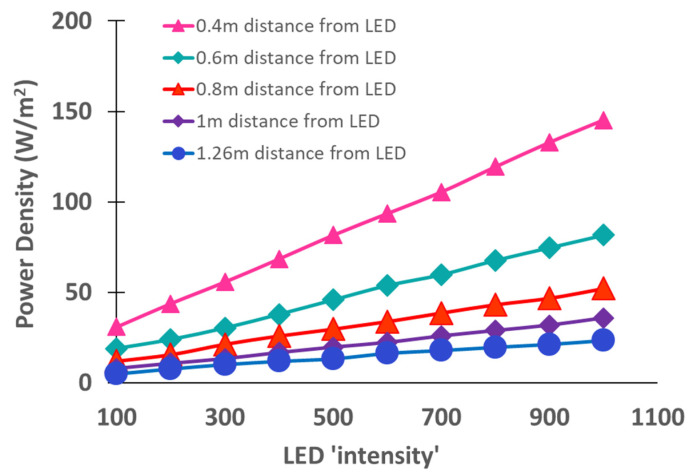
LED power density versus LED intensity for different distances from the LED source. The power density of the LED grows light is typically measured using a LaserCheck optical power meter to varying distances from the LED source.

It is important to note that an LED source has a relatively narrow bandwidth (~50 nm) and specific wavelength selectivity, making it ideal for indoor farming. For example, red LED illumination peaked at 630 nm can be applied in a horticultural application for regulating blossoming [31], whereas blue-violet LED can be used as a disease protection scheme [32]. However, excessive blue LED illumination typically generates oxygen radicals and causes photoinhibition in indoor farming. In addition to this, the intensity and photoperiod of the LED illumination are the most important key parameters that affect plant growth and quality [33]. For example, supplemental LED illumination (during day and night) can also overcome inherent plant shading and provide the required photoperiod, hence promoting early blooming. However, standard characterization is needed to optimize and control the LED illumination spectrum for maximizing the plant growth rate while maintaining high plant quality. Thus, it is necessary to calibrate the LED illumination source with an accurate characterization method before installing it. For example, a handheld laser power meter (LaserCheck, Coherent Scientific, South Australia) is typically used with a power measurement range between 10 µW and 10 mW at a wavelength range of 400 nm to 1064 nm. The power density (W/m^2^) of an LED illumination source can be calculated by measuring the output power of the LED source at a specific distance from the plant. Typically, the power density increases linearly with the LED intensity, but the slope of this relation is impacted by the distance from the LED source, as illustrated in Figure 2, which shows the LED power density versus LED intensity for different distances from the LED source.

In all cases, the effective use of LED illumination in terms of power consumption and uniform light distribution at the canopy level plays a vital role in plant photosynthesis. Typically, the LED illumination efficiency, which indicates the active power consumption, is calculated in terms of the illuminated leaves per unit power consumption (IPPC) as follows [34]:

$$\mathrm{IPPC}\;(µ\mathrm{mol}\;s^{-1}W^{-1})=\frac{\mathrm{Average}\;\mathrm{PPFD}\;({µ\mathrm{mol}\;s}^{-1}{\;m}^{-2}\times \;\mathrm{Projected}\;\mathrm{leaf}\;\mathrm{area}\;\left(m^{2}\right)}{\mathrm{Power}\;\mathrm{consumption}\;\mathrm{of}\;\mathrm{the}\;\mathrm{light}\;\left(W\right)}$$

Therefore, after quantifying the effect of the LED illumination and their interaction with the plant biology system. The next question would be: what steps should be taken to minimize the various losses in adequately capturing light in the plant canopy and identify the best light quality for the particular plant’s species? To address this question, experimental manipulations against controls are required in order to identify the daily photoperiod, the intensity of light and determine the optimum light spectrum that maximizes the yield of specific plant species and varieties.

The spectral composition of artificial light (light quality or spectral distribution of light) used in greenhouses usually deviates from natural solar light, thus causing long-term morphological and developmental changes, as well as short-term functional responses in plants. Light quality directly influences leaf photosynthesis via changes in stomatal aperture and photosynthetic quantum efficiency. Additionally, it depends on leaf characteristics, such as the size, number, and distribution of stomata over the upper and lower surfaces, which develop in the long term. Light quality-induced differences in stomal conductance are largely due to differences in stomatal density, which are mainly due to differences in epidermal cell size. Therefore, the leaf size is influenced by light quality and positively correlated with changes in stomal conductance across the applied light qualities [35]. 

One challenge with LED technology is that the color combination of red and blue LED illuminating the plant surface generates a purplish hue to the human eye, which can mask the visual identification of nutritional deficiency or any physical disorders. However, adding green light to a combination of red and blue LED illumination can resolve this problem [36].

Finally, iterative refining of experimental conditions and statistical analyses of alternative experimental treatments are required to reveal which treatment results in statistically significant differences in plant traits.

## 3. Photosynthesis

The arrays of light-emitting diode employed in the greenhouse environment can be a potential supplemental light source for the plant’s development. Its different light spectra could be tuned for particular plants requirements, thus improving the plant yield and profitability [37,38,39]. In general, red and blue light is essential for maximizing the photosynthesis process due to their strong absorption by the plant’s chlorophyll molecules. For example, the blue light is usually absorbed by cryptochrome, responsible for controlling the stomatal conductance and stem elongation. 

Plants typically produce carbohydrates through a chemical reaction, commonly called photosynthetic reaction (involving carbon dioxide, water, and light), whereby the light energy converts into chemical energy according to the following equation:

6CO_2_ + 6H_2_O + (light energy) → C_6_H_12_O_6_ (Sugar) + 6O_2_

Once a photosynthetic pigment is absorbed, the required amount of light and carbon dioxide enters the leaf stomata, and the photosynthesis process occurs. However, the pigment does not typically respond equally to all the wavelengths. For example, Chlorophyll A and Chlorophyll B react differently to different light qualities. Thus, increased leaf chlorophyll index in red pak choi and mustard has been observed under higher PPFD (545~440 μmol m^−2^ s^−1^) irradiance compared to a lower PPFD (220 μmol m^−2^ s^−1^) irradiance. This was first observed in 1972 when the photosynthetic efficiency curve measurement indicated that blue light is 25 to 35% less efficient than red light. In contrast, blue light is 5 to 30% more effective than green light in the photosynthetic process [40,41]. Besides, higher chlorophyll concentrations have been observed in tomato genotypes illuminated using LED modules (Philips, Eindhoven, Netherlands) with red (88% of intensity) and blue light (12% of intensity) at 662 nm and 456 nm peak wavelengths, respectively (150 μmol m^−2^ s^−1^) [14]. Increased leaf chlorophyll index in red pak choi and mustard has been observed with 545~440 μmol m^−2^ s^−1^ irradiance compared to 220 μmol m^−2^ s^−1^ irradiance [42,43]. Thus, plants exhibit a higher growth response when illuminated with blue and red wavelengths [44] and a lower growth response to UV, green, and infrared radiations.

The solar radiation impinging on plants can be modified using transparent cover materials or thin-film coatings [45]. Therefore, coating a clear glass panel with a thin film that passes only the blue and red wavelengths while blocking UV, green, and infrared radiation could accelerate the crop growth, leading to a higher crop yield. However, due to the unavailability of high PPFD light sources back in 1972, McCree et al. [40,41] measured the quantum efficiency curve using a low PPFD level within a short period of time. With the availability of high-power light sources, there is now a need to investigate the quantum efficiency curves of photosynthesis processes under high PPFD for several types of species. To date, insufficient quantitative data exist to perform a comparative analysis of the impact of LED illumination using quantum efficiency curves of photosynthesis processes.

## 4. Methodology for Comparative Analysis of LED Effects on Vegetables and Herbs

In order to review the effects LED illumination effects found on plants, we searched including peer-reviewed journals, books, and conference proceedings on the impact of LED illumination on plant growth and nutrient composition. To search the literature, we used Edith Cowan University’s (ECU) library search engine ECU Worldsearch [46] and relevant keywords, including “LED light effect on vegetable growth and nutrients”, “LED-based protected cropping”, and “LED effects on plant development and quality”. The words “vegetables” or “herbs” were kept constant to restrict the review to LED effects on vegetables and herbs. The initial search identified 205 studies. From this, 82 were removed due to not directly examining the impacts of LED illumination on morphological, physiological development, and nutrients on vegetables and herbs or not having sufficient quantitative data to extract for drawing valuable conclusions. Hence, this left 123 studies with quantitative measurements on plant responses. Only studies that provided quantitative data for treatments and controls were included. The collected data sets were screened based on physiological and nutrient parameters and extracted the dry mass, fresh mass, flavonoids, antioxidant activity, and phenolic compound. The reported data in each study was normalized for calculating the energy use efficiency using R cran [47].

First, the quantitative data were taken from the published paper (see Supplementary Table S1), then standardized and converted to the same scale (mg/g, g/day, %, etc.). Moreover, studies were separated based on wet and dry mass (g/day). Based on different studies, all the data (biomass, nutrients, energy use efficiency) were converted daily (per day). Only studies that compared control conditions to treatment (with LED) were included for the comparative analysis.

## 5. Effect of LED on Nutrients

Lighting conditions are one of the most critical factors affecting the photo-oxidative activity, which, in turn, alters the biochemical process in a plant, whereby antioxidative enzymes and bioactive compounds are accumulated. It prompts all plants’ photo-protective and growth processes and the metabolic light acclimation responses [48,49]. Thus, bioactive compounds and antioxidants synthesis in a plant could be triggered by using different LED wavebands, thus increasing the plant’s nutritional quality [50]. Therefore, adequate plant illumination is crucial for the greenhouse industry to maintain high-quality horticultural products [51,52]. However, little is known about the variations in the free-radical scavenging activities, the concentration of phenolic compounds, and the interaction between antioxidants in leafy vegetables under individual red light illumination [50]. Typically, in a plant, the nutrient captivation is not uniform [53]. Moreover, fewer nutrients are synthetized during the dormant period of the plants, whereas more nutrients can be captivated during periods of rapid vegetative growth [54]. Hence, recent research findings with the variation of LED illumination spectra on plants and changes its nutritional values open up a new and fruitful niche for future research efforts for greenhouse growers [51].

**Figure 3:**
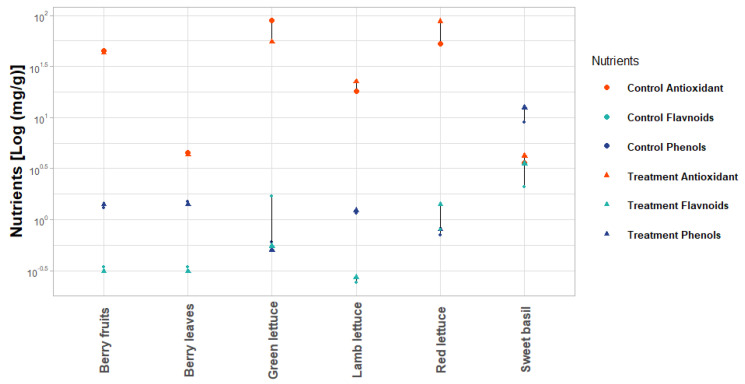
Impact of different LED illumination spectra on the nutrient’s parameters, namely, treatment antioxidant, treatment flavonoids, treatment phenols, treatment flavonoids, and control antioxidant, control flavonoids, control phenols for various vegetables and herbs in a grow chamber [55,56,57]. Plant traits were log-transformed due to significant variation in different treatments among species (phenols, flavonoids, antioxidant).

The comparative study identified six studies (lamb lettuce (*V. locusta* L.) [55], strawberry fruits (*F. ananassa*) [56], strawberry leaves [56], green lettuce (*Lactuca sativa* L.) [57], red lettuce (*Lactuca sativa* L.) [57], and sweet basil (*Ocimum basilicum* L.) [56]) with quantitative assessments of multiple secondary metabolites between two experimental conditions (control (without LED illumination) vs. treatment (with LED)). Overall, this revealed higher flavonoid content under LED in half of the antioxidant and estimates 50% of six studies and only one of the phenolic studies (Figure 3). Some secondary metabolites exhibited different responses across related plant varieties. For example, green lettuce displayed reduced flavonoid production under LED, while red lettuce showed increased production of flavonoids (Figure 3). Consistent patterns were observed for antioxidants, with green lettuce displaying reduced antioxidant content under LED, yet red lettuce displaying increased antioxidants (Figure 3). However, there were substantial differences in how individual metabolites respond, with antioxidants showing the most increase with LED treatment (2.6 mg/g of six studies) compared with flavonoids (0.7178 mg/g) and phenols (3.82 mg/g), which mostly showed reductions under LED conditions [55,56,57].

### 5.1. Antioxidant Properties

The antioxidant contents are typically monitored by measuring the DPPH (2.2-diphenyl-I-picrylhydrazyl) activity, which mainly refers to the ability to donate an electron to a hydrogen atom. The radical chain reaction primarily occurs in the antioxidant enzyme superoxide dismutase (SOD) that protects plants from oxidative stress. Superoxide (O^2−^) provides an extra electron reduction reaction to form H_2_O_2_ [58]. Photosynthetic pigments can absorb blue light more efficiently than other wavelengths, thus fixing the CO_2_ molecules, mainly in the upper palisade mesophyll. However, green light penetrates deeply into the plant. Hence, CO_2_ fixation occurs in the lower palisade and upper spongy mesophyll, and while this may involve synthesizing endogenous substances, it does not thwart the photosynthetic process [50]. 

Plants also absorb sunlight to produce secondary metabolites and high oxygen levels, making them enriched in antioxidants. The antioxidant and polyphenol contents can be stimulated by adding red light and blue light [15,44]. For varieties of baby leaf lettuces, it has been found that their nutritional quality is further affected by red and blue light [59,60]. Similarly, the red LED illumination (600 nm, 50 µmol m^−2^ s^−1^) has been found to augment the anthocyanin concentrations in red leaf cabbages [61] while adding supplemental far-red has an adverse effect on the nutrient contents. For example, far-red light (730 nm, 20 µmol m^−2^ s^−1^) with the combination of a red LED illumination (640 nm, 300 µmol m^−2^ s^−1^) suppresses the anthocyanin concentrations [20] by 40%, and also adding far-red light (700~800 nm) with 160 ± 5 μmol m^−2^ s^−1^ reduce the carotenoids concentration by 11%, compared to a single white light illumination [37]. 

Even red LED illumination supplementing HPS illumination (638 nm, 90 µmol m^−2^ s^−1^) alters the antioxidant activity and decreases parsley’s nitrate concentration [62]. Note that supplemental LED illumination helps accumulate phytochemicals in leafy vegetables [63]. For example, lettuce’s phytochromes are affected with the additional light treatment in preharvest stages, and their antioxidant capacity and phenolic compounds increase by 14.5% and 28.5%, respectively [64].

Anthocyanins can be considered phenolics and antioxidants, found in some vegetables and fruits [65], and are responsible for their vibrant coloring. The anthocyanin concentration can be increased by using supplemental 505 nm green LED illumination with an HPS lighting system for baby leaf lettuces (*Lactuca sativa* L.) [59]. Similar results can also be found in the case of lentil and wheat sprouted seeds that exhibit high antioxidant contents when supplemented with green (510 nm) LED spectra. In comparison, radish has the highest in antioxidants with supplemental amber (595 nm) light [66,67]. While, in the case of red cross baby leaf lettuces, anthocyanin and carotenoid concentrations can be increased (by 31 to 12%) by using UV-A LEDs as a supplemental light (373 nm, 18 ± 2 µmol m^−2^ s^−1^) for cool white fluorescent lamps [37]. Another study conducted by Samuolienė et al., 2012, has found that despite the decisive role of blue LED illumination in increasing the phenolic concentrations (by 28.5%) and antioxidant capacity (by 14.5%), it has adverse effects on the crucial dietary component such as decreasing ascorbic acid in baby leaf lettuces [59]. The same effect has also been observed under red LED illumination. For instannce, under 100% red light treatment at 660 nm (LEDs with 200 μmol m^−2^ s^−1^ at 16 h photoperiod), the ascorbic acid concentration in lamb lettuce decreases [55]. It is important to note that, although the antioxidant activity of vegetables and herbs is well studied, the exact source of the activity is not well understood. This might require a better understanding of the various genotype-environment interactions in vegetables and herbs [68].

### 5.2. Phenolic Compound

Phenolic compounds are naturally occurring secondary metabolites in vegetables, herbs, and fruits with varying quantities among the different parts of plants. Phenolic is one of the universal groups in bioactive plant compounds and is considered the most important secondary metabolite group [69]. Phenolic compounds function as antioxidants [70,71] and are vital secondary metabolites [72]. This is also responsible for taste, aroma, and color for vegetables and herbs [73]. In addition, they have several health benefits, including cancer prevention and anti-aging effect on the human body. 

Flavonoids are commonly known as phenolic compounds in almost all vegetables and herbs, mainly parsley and celery, which play a significant role in plummeting blood-lipid in the human body [74]. Thus, phenolic compounds are essential measures of the quality of vegetables and herbs, which a cultivation plant can stimulate under high-intensity LED illumination [75]. For instance, the use of supplemental red LED illumination (638 nm, ~170 µmol m^−2^ s^−1^) with an HPS (130 µmol m^−2^ s^−1^) lighting system increases the phenolic concentration of baby lettuce [37]. Higher phenolic and flavonoid compounds have been found under the white LED illumination and mix of LED illumination (white light control (W), white light with supplemental UV-A (WUV), white light with supplemental blue (WB), white light with supplemental green (WG), white light with supplemental red (WR) and white light with supplemental far-red (WFR)). All illumination spectra were recorded and averaged at five locations at the height of the plant canopy with a spectroradiometer (UV-A (350~400 nm), blue (400~500 nm), green (500~600 nm), red (600~700 nm), far-red (700~800 nm), PPF (400~700 nm), and red/far-red ratio are 11.5, 11.4 10.2, 10.7, 38.5 and 0.5 [37]) due to the primary metabolism enhancement, compared to using white LED illumination and far-red LED illumination. 

On the other hand, supplemental far-red illumination (700~770 nm) (LEDs with 185 μmol m^−2^ s^−1^] has been shown to increase the fresh weight as well as the bioactive compound in lettuce seedling [76]). Therefore, it is evident that a mix of LED illumination can be used to increase the phenolic compounds. For example, phenolics and flavonoids in Chinese foxglove can be increased by using blue LED illumination [77].

In contrast, red LED illumination improves the rosmarinic acid in basil plants [78]. However, it has no role in spinach phenols accumulation [62]. Similarly, green LED illumination negatively affects the accumulation of phenolic, flavonoid, and anthocyanin compounds in basil plants [79]. Using different supplemental LED spectra, Lee et al., 2018, demonstrated increased proliferation of total phenolics in harvested Chinese cabbages [80]. However, the adverse effect of red and blue light on nutrient accumulation has not been fully understood.

### 5.3. Essential Vitamins

Fewer vitamins are produced in plants if the sugar production in leaves stops due to ineffective photosynthesis processes. For instance, plants might become stressed under low-intensity light, resulting in vitamin deficiency at later growth stages [81]. However, during the photosynthesis process, the harmful side-effects of illumination intensity variation can be protected by the vitamin C existing in plants. Typically, the ascorbic acid (AsA) in plants acts as a regulator to scavenge reactive oxygen species (ROS), stimulate cell growth and division processes, and provide a precursor for oxalate. The human body is incompatible with synthesizing or accumulating ascorbic acid, and food, especially vegetables, which is the primary source of complementation of ascorbic acid. Biosynthesis and accumulation of ascorbic acid mainly depend on the illumination intensity and spectrum. A higher concentration of ascorbic acid in lettuce was observed with supplemental blue LED illumination or a mixture of red: blue illumination light than under red LED illumination. However, no significant difference under different LED light intensities was noticed [63,82]. Therefore, selection of the optimum intensity level is essential for obtaining the adequate photosynthetic photon flux necessary for (i) the adequate synthesis and production of photosynthetic pigments, (ii) canopy formation, and (iii) nitrogen initiation. It has been reported that, besides the morphological development of the plant, LED illumination can positively impact the production of vitamins and helps to reduce harmful compounds by adding green LED illumination as a supplemental light [83].

Similarly, red: blue (4:1) LED illumination (200 μmol m^−2^ s^−1^) promotes lettuce growth and decreases nitrate concentrations [84]. Another research has indicated that exposure to blue LED illumination significantly influences vitamin C concentration in cabbage [61]. In some cases, the effect of supplementary LED illumination on the contents of antioxidants and vitamin C is indirectly associated with the nitrate reduction rate [85]. For example, a significant decrease in nitrates was observed in all lettuce varieties under supplemental green (590 nm) light treatment [83,85]. Apart from the above findings, another extensive research carried out by Naznin et al., 2019 [44] has investigated the impact of LEDs on plant photosynthesis and greenhouse plant physiology, as well as the plant morphological response. They have found that blue light added with red light enhances the nutrient contents of lettuce, spinach, and kale [44,86] and increases the vitamin C concentration in tomatoes (G Samuolienė et al., 2010); however, it decreases the nitrate concentration [62,85]. Similarly, blue light at 460 nm peak wavelength has induced vitamin C in cabbage [86]. Simultaneously, a red LED illumination (638 nm, 90 µmol m^−2^ s^−1^) increases the vitamin C contents in several vegetable types, including rocket, mustard, and spinach [62]. These nutritional quality-related findings play a significant role in advancing greenhouse horticulture.

### 5.4. Carotenoids

Carotenoids are secondary metabolites, which act as critical aerial pigments that reduce the harmful effects of active threesome eminence of the chlorophyll molecules onto the photosynthesized components in the plant. The antioxidative effects of carotenoids, which consist of lutein and β-carotene, significantly lower the risk of some human diseases, including age-related eye diseases, lung cancer, and cardiovascular diseases. Vegetables are among the best natural sources of carotenoids [87]. Carotenoids also act as light-harvesting pigments in chloroplasts, protecting plants from photo-oxidative damage [88]. Similarly, in animals, both the lutein (LU) and zeaxanthin (ZEA) components of carotenoids have been shown to protect eyes from light-induced damage [89]. Illumination spectral components ranging from UV to far-red are the most critical factors influencing nutritional properties, such as concentration of carotenoids [90,91]. Lefsrud et al., 2008 have shown that the blue light and the ratio of red and blue light intensities help accumulate carotenoids (blue light 440 nm, 10.6 μmol m^−2^ s^−1^) [92]. Hydroponically cultured kale plants (*Brassica oleracea* L. *var. acephala D.C.*) were grown under specific LED wavelength treatments of 730, 640, 525, 440, and 400 nm had a maximum accumulation of chlorophyll A and chlorophyll B and lutein at the wavelength of 640 nm on a fresh mass basis. 

In contrast, b-carotene accumulation peaked under the 440 nm treatment [92]. However, this assumption needs to be confirmed through an extensive investigation of the effect of the ratio of red and blue light intensities for a wider variety of plant species. LU and ZEA compounds are found in the human retina as the main dietary carotenoids, and they improve the perception of the human eye [93]. Red-blue LED impacts LU and zeaxanthin’s content in broccoli microgreen [94] and red and green-leaf lettuces [95]. The carotenoid content also changes when adding red light as supplemental illumination. For example, pea seedlings illuminated with red LEDs for 96 h have increased the carotenoid contents [96]. A similar trend has also been encountered in parsley microgreens but not in basil microgreens, where the carotenoid counts have reduced with long red LED illumination periods [97]. A comprehensive study on leafy vegetables has shown that blue and red LED illumination have increased the carotenoid content in lettuces compared to using FL illumination only. In particular, the blue LED light was the dominant illumination responsible for the accumulation of carotenoids [95]. In addition, Q. Li and Kubota, 2009, have shown that the red LED illumination does not affect the carotenoid content, whereas the blue LED illumination increases the carotenoid content by 6 to 8% [37].

Interestingly, a notable increase in carotenoid concentration [98] has been observed in cabbage with an LED illumination flux of 300~400 µmol m^−2^ s^−1^. However, a similar carotenoid concentration has been attained with a flux of 125~300 µmol m^−2^ s^−1^ for kale, spinach, and mustard [99]. The use of supplemental red or yellow LED lights for 4 h/day has increased the carotenoid concentration in cucumber seedlings [100]. Sometimes, higher light exposure can create stress on the illuminated plant. In this case, it is crucial to accumulate higher carotenoids to protect plants from photo-induced effects. 

The effects of different illumination spectra (640 nm, 450 nm, and white) vary among vegetable species and cultivars. However, most leafy vegetables grown in controlled environments require a suitable range of photon flux (typically from 200 to 300 μmol m^−2^ s^−1^). In 2006, Lefsrud et al. demonstrated a very high accumulation of β-carotene and lutein contents with photon fluxes of 335 μmol m^−2^ s^−1^ and 200 μmol m^−2^ s^−1^ for kale and spinach, respectively [92]. Blue or yellow LED illumination affects the rates of respiration and ethylene initiation of harvested fruits and vegetables, and this not only improves the synthesis of β-carotene, lutein, α-tocopherol, and γ-tocopherol but also enhances fruit ripening.

It has also been found that different lighting conditions and the duration of supplemental illumination can adversely affect a specific plant and its biochemical synthesis pathways. For example, for green vegetables, short-duration treatment by supplemental red LED illumination has been less functional in enhancing antioxidants and nutritional components, resulting in decreased nutritional quality [62]. This, however, is inconsistent with the results reported by Q. Li and Kubota, 2009, which show that supplemental LED illumination of different spectra (UV-A, blue, red, far-red, or their ratios) should be applied more strategically, recommending further investigation to be carried out to fully understand the biochemical synthesis pathways over the growth stages of vegetables [37].

## 6. Effect of LED on Physiology

Photomorphogenesis is the second most discussed process after the photosynthesis process. It is facilitated by a photoreceptor and considered a default process for developing vegetables and herbs, where phytochromes play a crucial role in response to light signals. Photomorphogenesis has been investigated by several research groups, concluding that the LED illumination spectrum has a high impact on plant physiology and growth morphology [101,102]. For instance, several experiments have been conducted over the last decade, revealing that the blue, red, far-red, and green LED illumination bands strongly affect the growth and nutritional quality of vegetables. The leaves and shoots are the leading edible parts of vegetables and herbs affected by the LED illumination. For instance, wavelength-specific findings have revealed that the seedlings were first grown in blue LED illumination for 30 days (blue 460 nm, LEDs with 80 μmol m^−2^ s^−1^ and then transferred to sunlight (normal level of 350 μmol m^−2^ s ^−1^) at the 31st day and cultured for 30 days (60 days) to sunlight with 350 μmol m^−2^ s^−1^) [86]. It has been observed that blue LEDs benefit vegetative growth while red LEDs and blue plus red LEDs support reproductive growth in non-heading Chinese cabbage [86]. 

The increase in leaf area is one of the most important physiological parameters used to estimate the effective photosynthetic rates per unit area. For example, it has been reported that the largest leaf area is typically produced under blue LED illumination [15,103]. Note that a large leaf area allows a higher light-plant interception [37]. Similarly, using supplemental blue LED illumination in conjunction with red LED illumination (95% R + 5% B, LEDs with 200 ± 5 µmol m^−2^s^−1^) has a positive influence on the growth of lettuce, spinach, and kale, producing a higher number of leaves per plant [44]. However, reduction in leaf area has been observed for cannabis Sativa grown under blue and red LED illumination (100% blue + red light or 50% white LED light, 200 ± 30 µmol m^−2^s^−1^). This was possibly due to the uncontrolled growth conditions and variations in the spectrum of the light source.

Fresh weight is one of the most significant development indicators for vegetables and herbs. It depends upon the spectrum of the light applied and the types of grown vegetables and herb species. For example, detailed investigations conducted by Hogewoning et al., 2010 [104] and Samuoliene et al., 2012 [105] have shown that blue LEDs supplemental with red LEDs or high-pressure sodium lamps, significantly increased fresh weight in cucumber (blue LEDs (450 nm) additional to red (638 nm) light with a total PPFD of 100 ± 5 μmol m^−2^ s ^−1^) [104] and tomato plants (blue LED with 15 μmol m^−2^ s ^−1^ and HPS lamps was about 90 µmol m^−2^ s ^−1^) [105]. On the other hand, Fanwoua et al., 2019 have found that the fresh weight of tomato slightly increases under red/blue light and far-red light (peak at 739 nm) (LEDs with 200 µmol m^−2^s^−1^ + 40 µmol m^−2^s^−1^) [106]. 

The dry mass (biomass) is typically measured after the plant is sequentially dried in a drying oven and is an indicator of plant biomass content and the quality of growth. LED illumination has also been shown to have a positive impact on biomass build-up. For example, 26.06% of dry mass has accumulated under 10B: 90R LED illumination [107]. Similarly, the dry weight of basil shoots is highest under 30B: 70R LED illumination (LEDs with 250 ± 10 µmol m^−2^ s^−1^) [108]. Similar results have also been reported in Piovene et al., 2015, where the lateral shoot fresh weight for basil plants is highest under monochromatic blue LED illumination (when 100% blue light increased from 9 to 18 μmol m^−2^s^−1^) [56]. While the biomass, root diameter, and root volume increase in radish plants under 2R:1B with 16 h/8 h photoperiod (LEDs with 200 to 300 µmol m^−2^s^−1^) [109]. Similarly, a higher biomass yield has been observed in coriander under 10:1 (red: blue) at 661 and 449 nm peak wavelengths, respectively (LEDs with 120 μmol m^−2^s^−1^) [110]. This biomass yield confirms the importance of the intensity and quality of illumination for satisfactory plant development. Thus, it is evident that the blue LED illumination promotes multiple desirable plant growth factors. Hence, it has a reasonable (and possibly under-explored) potential use in plant production, especially in greenhouse environments.

The photosynthetic pigments of plants absorb the visible light effectively, and experiments have shown that red LED illumination (red light, 600~700 nm, LEDs with 360 ± 10 μmol m^−2^ s^−1^) to blue illumination improves the yield of tomatoes [111]. On the other hand, a study conducted by [44] has shown that using 100% red LED illumination negatively impacts the growth of spinach, lettuce, and sweet basil compared to a combination of red and blue LED illumination (91% red + 9% blue, by the photon counts). Moreover, additional red LED illumination (635 nm, and 660 nm, with 170 ± 10 μmol m^−2^ s^−1^) has been shown to delay the transition to blossoming in basil and Indian mustard [112], and this is attributed to the “red-light syndrome” phenomena. The second essential growth indicators for vegetables and herbs are height and stem diameter. When cultivated under red and blue illumination, monkey-pepper (*Piper aduncum* L.) plants have attained the highest stem diameter [113]. Similar results have been observed for green and purple basil plants, with the thickest stems followed under red (600~700 nm). In contrast, under blue LED illumination, the stems were the thinnest (400~500 nm). On the other hand, the average shoot dry weight was highest for a combination of red and blue illumination (70% R + 30% B light, LEDs with 250 ± 10 µmol m^−2^s^−1^) [108]. Thus, red LED illumination plays a significant role in optimizing morphological development to maximize crop yield and improve its quality.

Lettuce’s fresh weight has been reported to increase under additional far-red LED illumination (wavelengths between 700 to 800 nm) [37,76,101,114]. Lee et al., 2016 [76] and Pinho et al., 2009 [115] have both reported that far-red LED illumination increases the total biomass and plant elongation for lettuce [114]. However, at the same time, far-red LED illumination decreases the pigment concentration of lettuce [37], which negatively affects the plant quality at later plant development stages. Interestingly, a green LED has recently increased the fresh weight of lettuce [116] and cucumber transplants [105]. Several studies have also suggested that supplemental green LED illumination can improve the quality of tomato and sweet pepper [42,62]. Similar results have confirmed that the addition of green LED illumination accelerates the growth of lettuce [15,116]. However, the mechanism behind the greenlight effect is not comprehensively explored yet. Without proper scientific insights, an increased growth rate for some species has been observed by adding green LED illumination power of up to 24% of the overall illumination power (by photon energy counts) [117]. Recently, the Bioresource Engineering Group at McGill University, Canada [118] has found that using only green LED illumination is not adequate for achieving optimum plant growth. However, green illumination might improve the plant’s major morphological parameters with other LED illumination inputs. This way, a green LED illumination could help to promote the desirable factors in crop production.

**Figure 4:**
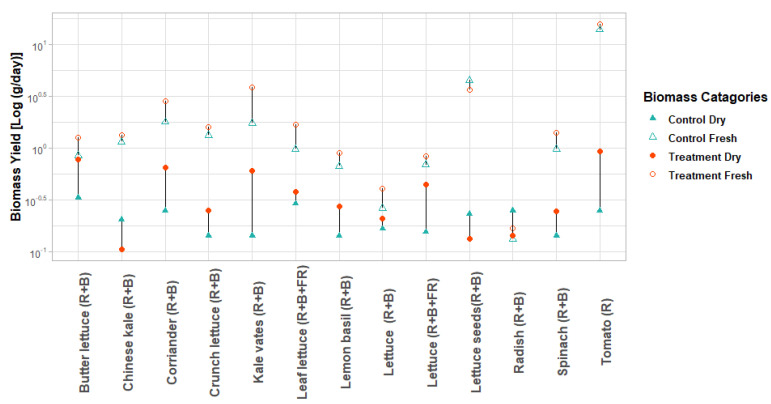
Effect of different LED illumination spectra on plant growth and development parameters, treatment fresh mass and treatment dry mass (with LED illumination), control fresh mass, and control dry mass (without LED illumination) for different vegetables and herbs grown in the growth chamber. Plants traits were log-transformed due to considerable variation in different treatments among species.

In this study, the comparative analysis identified 13 studies (such as red leaf lettuce (*Lactuca sativa* L.) [15], buttercrunch lettuce (*L.S.var. capitata*) [44], kale-vates (*Brassica oleracea*) [44], lemon basil (O. *basilicum citriodorum*) [44], spinach “Unipack 151” (*Spinacia oleracea*) [44], red butter lettuce (*Lactuca sativa* L.) [57], butterhead lettuce (*Lactuca sativa var. capitata*) [84], cherry radish (*Raphanus sativus* L.) [109], coriander leisure (*C. sativum*) [110], tomato (*Solanum lycopersicum* L.) [111], Chinese kale (*Brassica alboglabra Bailey*) [119], lettuce (*Lacuta sative* L. “*Michalina*”) [120], and lettuce seeds batavia diablotin (*Lactuca sativa* L.) [121], that reported quantitative data related to LED illumination on biomass yield with two experimental conditions control vs. treatment. The review revealed that LED illumination significantly increased average biomass yield (g/day), ranging from 39.55 to 95.95% of the 13 species for dry mass and fresh mass, respectively (Figure 4). These differences range from a 2-fold difference to a 9-fold difference in yield for both categories.

## 7. Effect of LED on Postharvest Quality and Resource Use Efficiency

The primary postharvest aim is to prevent deterioration of fruit and vegetables and minimize spoilage from microorganisms to optimize their commercial value [122]. The application of LED illumination for increasing the storage life of vegetables and herbs has attracted great interest. For example, it has been shown that the storage life of leafy vegetables increases with low-intensity illumination compared to storing them in a dark place [123]. In addition, to maintain optimal postharvest species quality, other critical conditions parameters such as temperature, relative humidity, and the CO_2_, ethylene, and O_2_ levels need to be optimally controlled [124]. Several postharvest studies have shown that nutrients typically increase when exposed to fluorescent light sources [125]. 

However, recently, LEDs have emerged as potential postharvest light sources. Due to their higher moisture content, vegetables and herbs typically have a short shelf life, making it challenging to store them for extended periods. Vegetables and herbs are also prone to microbial degradation, which accelerates the degradation of their nutritional quality [126]. It has been shown that light treatment with optimal intensity and spectrum is essential for the maximization of the shelf time of vegetables and herbs [127]. Therefore, it is essential to store vegetables and herbs using higher optical power intensities and optimum illumination spectra. For instance, UV-A LED illumination has been shown to have a positive postharvest impact. However, UV-A LED sources typically have low output optical power, making them impractical for vegetables and herb storage [128]. However, excessive light typically induces photo-oxidative stress on the plant, and that reduces the postharvest quality [129]. Note that optimizing LED illumination spectrum can be far effective than using pulsed illumination. For instance, white LED illumination with low PPFD delays the aging of lettuces, increases chlorophyll, and slowly reduces the pheophytin level [123]. It has also been found that with a combination of white and blue LED illumination, the postharvest yellowing (aging) of broccoli was significantly lowered compared to postharvest storage in dark places [130]. Moreover, the use of supplemental LED illumination in conjunction with traditional light sources has been shown to increase the preharvest nutritional quality of vegetables and herbs [63]. For example, green and white LED illumination increases the chlorophyll content compared to red and blue LED illumination [131], while the vitamin C and phenolic concentrations are highest with blue and white LED illumination. It is important to note that high-intensity illumination typically increases vegetables and herbs’ transpiration, hence reducing their biomass and quality. For example, blue light illumination increases leaf transpiration (Muneer et al., 2014), thus affecting the consumer’s acceptance of the vegetables and herbs. Interestingly, leaf transpiration can be significantly reduced by using IR LED illumination [132]. Therefore, the incorporation of IR LED illumination with other adequate illumination spectra could minimize postharvest water loss, and optimize the energy use efficiency. 

**Figure 5:**
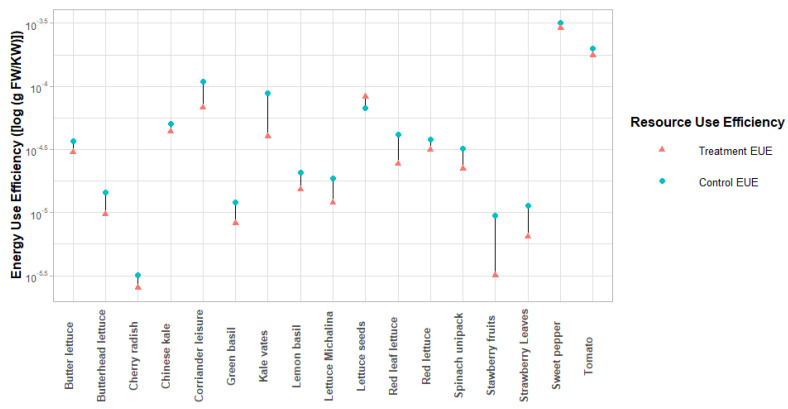
The energy use efficiencies (EUE) were calculated for vegetables and herbs grown in growth chambers, with (treatment) and without LED illumination (control) [15,44,57,84,109,110,111,119,120,121].

The energy use efficiencies (EUE) is defined as the average biomass produced per unit of electrical energy used for plant illumination [133]. Figure 5 shows the effect of LED illumination treatment on the energy use efficiency (EUE) values for 17 vegetable and herb species grown in the grow chamber.

**Figure 6:**
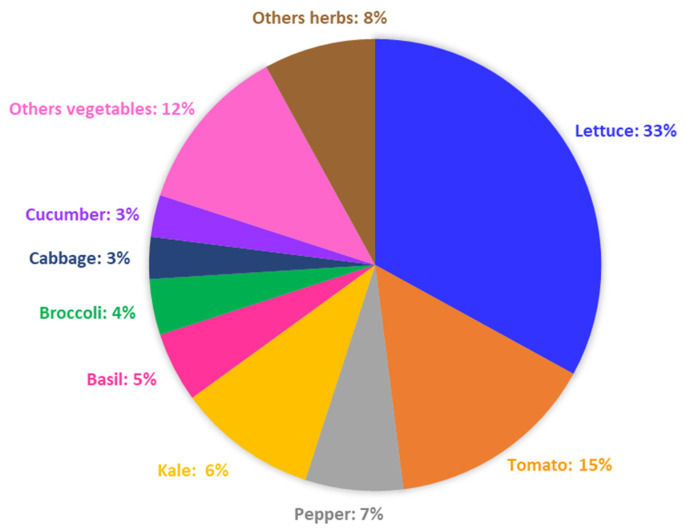
The pie chart shows the relative percentage of published research reported in the literature and used in this review for the different types of vegetables and herbs treated with LED illumination.

The EUE is a crucial metric to quantify the fresh mass produced per unit of electrical energy used for plant illumination. The comparative analysis of vegetable and herb species grown in the growth chamber revealed that across 17 species displayed higher energy use efficiency using LED illumination (Figure 6). The increase in EUE was most significant in strawberry fruits (194.87%) and the smallest in sweet pepper (10.15%). These differences highlight the varied response to LED across different species and varieties; thus, making broad generalizations is difficult.

## 8. The Implication of LED on Plants Development and Its Future Policy

This paper described the outcome of the study on the effect of LED illumination on vegetables and herbs over a dozen plant species and varieties. Lettuce was the dominant greenhouse-grown vegetable encountered in this study, accounting for about 33%, while reports on cabbage were few, accounting for only 3% (Figure 6). Lettuce grows better in an indoor environment than outdoor, and it can be harvested quickly, while cabbage grows very slowly and needs a broader space for cultivation. After lettuce, tomato, pepper, kale, basil, and broccoli represented 15%, 10%, 7%, 5%, and 4% of the reviewed article on vegetables and herbs affected by LED illumination (Figure 6). Other vegetables (e.g., potato, radish, etc.) accounted for only 12% of the reviewed papers. 

In this review, red LED illumination (45%) and blue (40%) LED illumination are the primarily accessible light spectra for indoor-grown plants. The red LED illumination is the most commonly used light in greenhouse-grown plants (as it is the dominant source responsible for photosynthesis). In contrast, the far-red (5%) LED illumination only allows for plant-specific applications. Hence, it is not commonly used in greenhouse applications. Even though far-red LED illumination has many benefits, such as compensating for shading, this light spectrum has not been widely used, possibly because (i) it is relatively newly assembled in commercial LED fixtures and (ii) there is no reliable data that confirms the significance of far-red illumination for the maximization of the plant nutritional quality.

Over the last decade, LED-based illumination systems have become very popular in the horticulture community. However, the high energy costs associated with supplemental lighting are the main limiting factor in deploying additional lighting in horticultural greenhouses [134]. Another factor limiting the use of LED illumination in horticultural greenhouses is the consumer reluctance to accept new technologies over classical cultivation approaches [135]. In addition to their high costs and lack of consumer acceptance, LED illumination sources have not been widely adopted because there is no single universal illumination spectrum recipe for all plants [136]. 

Moreover, white LED sources have been produced using blue LEDs with phosphor coatings (phosphor absorbs some blue light and converts it to broadband yellow light) [137]. Other issues that need to be addressed are the importance of green and far-red illumination for efficient plant growth and photosynthesis. Another critical wavelength is far-red. Park and Runkle [138] have found that supplementing red and blue LEDs with far-red accelerates plant growth through the excitation of the photosystem, which increases the photosynthetic efficiency and improves the overall rate of photochemistry and CO_2_ assimilation [139]. However, foliar intumescence may develop without the ultraviolet spectral component by using a single-color illumination [140]. This foliar intumescence is a severe problem for some crops, which needs to be investigated to adopt artificial lighting in greenhouses [38]. As we have discovered in our comparative analysis of the literature, the general conclusion regarding LED-based plant growth systems is that the plant response to LED illumination is species-specific [141]. It is also generally accepted that the mostly adopted illumination spectra comprise only the red and blue (and sometimes far-red) spectral components is, not the whole PAR range [142]. Therefore, close collaboration between LED manufacturers and horticulture growers is expected to produce cost-effective universal LED illumination sources [143]. This collaboration will also trigger innovative and fundamental research within the scientific community, thus finding versatile illumination spectral recipes for various plant species. Overall, there is a need to improve consumer perception about LED illumination and establish synergy between growers and the industry to drive optimal LED illumination sources for horticultural production in greenhouses.

## 9. Summary and Suggestions

The fresh and dry mass of the vegetables and herbs can be increased by increasing the intensity of red-light irradiation, which stimulates the growth of leafy vegetables. On the other hand, the compactness and elongation of plants can be improved through supplemental blue illumination. Blue illumination contributes to several desirable factors, such as plant compactness, vegetative growth, and potentially higher pigment and concentration of vitamin C. Thus, blue illumination is desirable in plant production. According to reported results, red illumination increases the yield by decreasing the nitrate and improving plants’ vitamin C. These two factors are necessary for crop quality; therefore, red illumination is essential and has a significant role in improving plant quality. As plant elongation and low pigment concentration are not always desirable, far-red illumination may not be crucial for increasing the plant biomass (at least for some plants). Since green illumination accelerates plant growth and increases the nutritional quality (e.g., vitamin C), thus the green wavelength is necessary for maximizing crop yield. Further investigations are still needed to confirm the impact of green and far-red illumination on a plant to improve the biomass and nutrient contents of vegetables and herbs. Therefore, the outcomes of different percentages of light response on plant growth and nutritional quality could be used to design the optimized lighting system for various horticultural greenhouse applications. 

Arbitrary control of the intensities of LED illumination spectral components enables the continuous optimization of the illumination spectrum over time, typically required in real-life scenarios. For example, far-red can be an effective non-chemical means to control plant morphology [144]. However, with short exposure to far-red, the mechanisms responsible for increasing the plant biomass are still not well explored. They may be attributed to hormonal changes and transpiration. Cope et al., 2013 indicated that the optimal illumination spectrum for plant growth is likely to change with the plant age and stage and environmental conditions [145].

In some experiments, LEDs have been used as supplemental light sources in conjunction with sunlight. For example, some solar spectral components might be attenuated during the day, hence insufficient for optimal plant growth [146]. In most cases, larger plants intercept a relatively large portion of the sunlight, thus becoming longer than other plants. In this case, the canopy light interception capacity can be made uniform by using supplemental LED illumination. 

However, due to their high initial installation costs, LED illumination application in agricultural systems is limited to small-scale facilities [30]. Research and development activities on the fabrication of cost-effective LED lighting systems have varied rapidly over the last few years. Thus, the cost of LED lighting systems is expected to be reduced significantly. For example, the efficiency of blue LED illumination sources rapidly increased from 11% in 2006 to 40% in 2011 [147], and currently, it exceeds 75%. On the other hand, modern greenhouses equipped with LED illumination sources and energy-efficient materials are expected to have low production costs and energy consumption [148,149]. Ongoing LED technology development has broadened the scope of LED lighting systems in horticultural greenhouses and hygienic storage, even in developing countries.

Therefore, LED illumination spectrum manipulation could enable significant morphological adaptations, and identification of the wavelength ranges is required to increase the plant photosynthesis process. However, far-red and green LED lighting imposes several challenges, such as illumination with more than 50% of green LED light results in a shorter plant. In contrast, far-red light has a “shade avoidance response” since typically plants elongate in an attempt to capture the available light. In some cases, an elongation response is desirable, except in the production of ornamentals. However, studies do expose scenarios under which red LED illumination is not beneficial for specific cases. For example, using red light solely is not always favorable for plant progression due to the “red light syndrome”. However, it is reported that supplemental red LED illumination can help accumulate bioactive compounds and phenolic compounds, and antioxidant properties of the vegetables and herbs. Therefore, this paper thoroughly reviewed the effects of the LED illumination spectrum on the plant’s morphological development, bioactive compound, and antioxidant properties.

## 10. Conclusions

The use of LED illumination sources for increasing the growth rate of greenhouse-grown vegetables and herbs is on the rise worldwide. This massive attractiveness of LED is due to the exponential increase in LED installations in greenhouses, positive changes in policies on reducing energy consumption, and its minimal negative impacts on the environment. However, further research is still required to fully understand the effect of the LED illumination spectrum on plant growth and the associated optobiological interactions governing the photosynthesis process. As more durable and cost-effective LED sources with a diverse range of wavelengths continue to emerge in the market, the adoption of LED illumination sources in greenhouses is expected to accelerate, leading to better horticultural production yields with a short cultivation cycle. This review has highlighted that the valid ranges of wavelength identified in the literature are red (640~720 nm), blue (425~490 nm), green (490~560 nm), and far-red (~720–750 nm), which are considered the most valuable wavelengths due to their direct and indirect impacts on plant growth and quality. 

LED spectral characterization methods have been presented in this review, suitable for examining the next-generation LED technologies desired for deployment in protected cropping facilities, which enable high crop yield and quality to be attained cost-effectively. It is essential to mention that the work presented in this review paper predominantly covers widely used red, blue, green, and far-red LED illumination sources for greenhouse and growth chamber grown plants. It excludes other wavelength ranges, such as UV, yellow, and orange, which have rarely been deployed for growing vegetables and herbs.

Overall, optimizing the LED illumination spectra in greenhouses for crop yield and quality improvement addresses food security and the cost-effectiveness of deploying LED sources in large-scale greenhouse installations. However, significant hurdles remain in the widespread deployment of LED illumination and in developing efficient, cost-effective LED-based greenhouses. Consequently, significant advances will most likely emerge from multidisciplinary research and development activities involving close collaboration amongst LED manufacturers, greenhouse growers, researchers, and government stakeholders.

## Figures and Tables

**Table 1:** Technical terms and abbreviations are used in the article.

Abbreviation	Definition	Units
PAR	Photosynthetic Active Radiation	μmol m^−2^ s^−1^
PPF	Photosynthetic Photon Flux	μmol s^−1^
PPFD	Photosynthetic Photon Flux Density	μmol m^−2^ s^−1^
FPAR	Fraction of Photosynthetically Active Radiation	NA
IPPC	Illuminated Leaves Per Unit Power Consumption	µmol s^−1^ W^−1^
Power Density	Power Transferred Per Unit Area	W/m^2^

## Supplementary Materials

The following are available online at https://www.mdpi.com/article/10.3390/plants10112470/s1, supplementary Table S1: physiological and nutritional properties studies in the article.
