# Supplementary material for: LED Illumination for High-Quality High-Yield Crop Growth in Protected Cropping Environments
Source: Plants (Basel). 2021 Nov 16;10(11):2470. doi: 10.3390/plants10112470 (PMC8621602; doi:10.3390/plants10112470)
Supplement: Supplementary file 1 [file plants-10-02470-s001.zip › plants-1405523-supplementary.pdf]

Supplemental

Table S1. Physiological and nutritional properties studies in the article

Plant	PPFD (μmol $m^{-2}s^{-1}$)	Energy Density (W/m^2)	Energy Use Efficiency (g FW/KW)	Daily light Integral (DLI)	Treatment Dry Mass (g/day)	Treatment Fresh Mass (g/day)	Treatment Plant Height (cm/day)	Treatment Leaf Number	Treatment Antioxidant Activity (%)	Treatment Phenols (mg/g DM)	Treatment Flavonoids (mg/g)	Treatment Carotenoids (mg/g DM)	Treatment Total Ch (mg/g)	Treatment Anthocyanin Contents (mg/g)	Treatment PPFD (μmol $m^{-2}s^{-1}$)	Control Dry Mass (g/day)	Control Fresh Mass (g/day)	Control Plant Height (cm)	Control Leaf Number	Antioxidant Activity (%)	Control Phenols (mg/g DM)	Flavonoids (mg/g DM)	Control Carotenoids (mg/g DM)	Control Total Ch (mg/g DM)	Control Anthocyanin Contents (mg^{-1})	Reference
Spinach unipack 151	200	43.763676	11.69847719	11.52	0.247551	1.402653061	0.726530612	72.5	63	NA	NA	0.21996	0.77739	NA	200	0.14183674	0.97346939	35.17	36.33	60	NA	NA	0.16372	0.60238	NA	Naznin at al., 2019
Lettuce 'Michalina'	100	21.881838	6.751630951	5.04	0.005	0.404761905	0.5	NA	NA	3.96	6.28	0.082	0.206	NA	100	0.16547619	0.26190476	21	NA	NA	4.451	6.28	0.057	0.345	NA	Sergejeva at al., 2018
Sweet Pepper	200	43.763676	116.4043586	11.52	6.2344898	13.95693878	10.04632653	191.61	65	NA	NA	0.29592	0.85977	NA	200	0.14183674	12.6697959	459.42	161.39	66	NA	NA	0.17142	0.68685	NA	Naznin at al., 2019
Green basil	250	54.704595	NA	14.4	0.0393333	NA	0.766666667	NA	NA	NA	NA	NA	1.6	0.75	250	0.23166667	NA	17	45	NA	NA	NA	NA	0.8	0.65	Hosseini at al., 2019
Cannabis sativa	200	43.763676	NA	12.96	NA	NA	1.107142857	NA	NA	NA	NA	NA	NA	NA	200	0.24821429	NA	35	NA	NA	NA	NA	NA	NA	NA	Lalge at al., 2017
Buttercrunch lettuce	200	0.0437637	36.58331633	11.52	0.2506122	1.601020408	0.394489796	26.67	55	NA	NA	0.20802	0.80039	NA	200	0.14183674	1.32285714	24.33	24.67	55	NA	NA	0.15172	0.66138	NA	Naznin at al., 2019
Cherry radish	240	52.516411	1.166642113	13.824	0.0142857	0.167857143	NA	3.3	NA	NA	NA	NA	NA	NA	240	0.24821429	0.13214286	NA	3.7	NA	NA	NA	NA	NA	NA	Zha at al., 2018
Tomato seeds	150	32.822757	NA	6.48	0.8214286	NA	5.357142857	NA	NA	NA	NA	0.0028	0.01798	NA	150	0.24821429	NA	120	14	NA	NA	NA	0.00314	0.02132	NA	Kalaitzoglou at al., 2019
Butterhead lettuce	400	87.527352	5.248400178	17.28	0.077619	1.258571429	NA	NA	NA	1.55	NA	0.13	NA	NA	400	0.33095238	0.84761905	NA	NA	31	1.44	NA	0.13	NA	NA	Bian at al., 2016
Corriander leisure	120	26.258206	39.7154762	6.912	0.6428571	2.857142857	1.714285714	7	65	NA	NA	NA	NA	NA	120	0.24821429	1.78571429	50	5	35	NA	NA	NA	NA	NA	Naznin at al., 2016
Kale vates	200	43.763676	32.33123443	11.52	0.6063265	3.876530612	0.574897959	28.17	66	NA	NA	0.26123	0.96839	NA	200	0.14183674	1.74387755	51.63	29.17	65	NA	NA	0.22328	0.75726	NA	Naznin at al., 2019
Red leaf lettuce	185	40.4814	15.05377556	7.992	0.0675	1.669583333	NA	12	13	12	NA	NA	0.016	NA	185	0.28958333	0.98083333	NA	13	NA	NA	NA	NA	NA	NA	Lee at al., 2016
Vito cultured potato	100	21.881838	11.27125214	5.76	0.2778571	0.675714286	NA	NA	NA	NA	NA	NA	2.81	NA	100	0.24821429	14.3003571	NA	NA	NA	NA	NA	NA	2.74	NA	Chen at al., 2020
Tomato genotype	150	32.822757	NA	8.64	0.952381	NA	1.095238095	7	NA	NA	2	NA	2.2	0.12	150	0.33095238	NA	28	6	NA	NA	0.12	NA	1.7	0.17	Ouzounis at al., 2016
Lamb lettuce	200	43.763676	NA	11.52	0.1373667	NA	NA	NA	22.47	1.2227	0.2699	NA	NA	NA	200	0.11583333	NA	NA	NA	18.26	1.1553	0.2421	NA	NA	NA	Wojciechowska at al., 2015
Chinese kale	120	26.258206	18.35672672	5.184	0.1058824	1.320588235	0.75	15	NA	NA	NA	0.21	1.76	NA	120	0.20441177	1.15	22.8	13.2	NA	NA	NA	0.16	2.06	NA	Liu at al., 2020
Stawberry fruits	200	43.763676	3.425459822	11.52	NA	0.410714286	NA	NA	43	1.4	0.34	NA	NA	NA	200	0.12410714	0.13928571	NA	NA	45	1.3	0.34	NA	NA	NA	Piovene at al., 2015
Strawberry Leaves	200	43.763676	4.155231697	11.52	NA	0.498214286	NA	NA	4.3	1.5	0.34	NA	NA	NA	200	0.12410714	0.28214286	NA	NA	4.5	1.3	0.34	NA	NA	NA	Piovene at al., 2015
Lemon basil	200	43.763676	7.528352193	11.52	0.2744898	0.902653061	2.193877551	107.5	70	NA	NA	0.29592	0.85977	NA	200	0.14183674	0.66693878	51.12	90.5	70	NA	NA	0.17142	0.68685	NA	Naznin at al., 2019
Sweet basil	200	43.763676	13.07535968	11.52	NA	1.567741935	NA	NA	4.2	12.5	3.5	NA	NA	NA	200	0.22419355	1.64193548	NA	NA	3.6	8.9	2.1	NA	NA	NA	Piovene at al., 2015
Tomato	360	78.774617	72.9771875	20.736	0.9214286	15.75	NA	NA	NA	NA	NA	NA	NA	NA	360	0.24821429	13.8571429	NA	NA	NA	NA	NA	NA	NA	NA	Lu at al., 2012
Red lettuce	100	21.881838	13.92636411	5.04	0.0443333	0.834888889	NA	17	NA	0.1383	NA	0.22	0.0064	0.13	100	0.15444444	0.68888889	NA	NA	NA	0.1388	NA	0.276	0.0084	0.11	Johkan at al., 2010
Green lettuce	250	54.704595	23.63070833	9	0.1458333	3.541666667	NA	NA	55	0.5	0.55	0.22	1.25	NA	250	0.28958333	NA	NA	NA	90	0.6	1.7	NA	1.05	NA	Li at al., 2020
Red lettuce	250	54.704595	16.12448333	9	0.1145833	2.416666667	NA	NA	88	0.8	1.4	0.18	1.7	NA	250	0.28958333	NA	NA	NA	53	0.7	0.8	NA	1.7	NA	Li at al., 2020
Lettuce seeds	250	54.704595	24.46473333	14.4	0.1333333	3.666666667	NA	28	NA	NA	NA	0.0124	0.057	0.2	250	0.23166667	4.5	NA	22	NA	NA	NA	0.0117	0.039	0.15	Camejo at al., 2020
Pak choi	130	28.44639	1.64971978	7.488	0.015	0.128571429	NA	NA	NA	NA	NA	NA	NA	NA	130	0.49642857	1.42857143	NA	NA	NA	NA	NA	NA	NA	NA	Bian at al., 2018
Tomato solanum	150	32.822757	NA	12.96	NA	NA	NA	NA	4.26	1.7	NA	NA	0.4	NA	150	0.49642857	NA	NA	NA	3.25	1.34	NA	NA	0.7	NA	Bian at al., 2019
Sweet green basil	215	47.045952	4.376517591	12.384	NA	0.564102564	NA	NA	0.9	NA	1.6	NA	NA	NA	215	0.17820513	0.38461539	NA	NA	0.7	NA	0.9	NA	NA	NA	Pennisi at al., 2019
